# Metal-Insulator Transition in Nanoparticle Solids: Insights from Kinetic Monte Carlo Simulations

**DOI:** 10.1038/s41598-017-06497-1

**Published:** 2017-08-01

**Authors:** Luman Qu, Márton Vörös, Gergely T. Zimanyi

**Affiliations:** 1 Physics Department, University of California, Davis, USA; 2 Materials Science Division, Argonne National Laboratory, Lemont, IL 60439 USA

## Abstract

Progress has been rapid in increasing the efficiency of energy conversion in nanoparticles. However, extraction of the photo-generated charge carriers remains challenging. Encouragingly, the charge mobility has been improved recently by driving nanoparticle (NP) films across the metal-insulator transition (MIT). To simulate MIT in NP films, we developed a hierarchical Kinetic Monte Carlo transport model. Electrons transfer between neighboring NPs via activated hopping when the NP energies differ by more than an overlap energy, but transfer by a non-activated quantum delocalization, if the NP energies are closer than the overlap energy. As the overlap energy increases, emerging percolating clusters support a metallic transport across the entire film. We simulated the evolution of the temperature-dependent electron mobility. We analyzed our data in terms of two candidate models of the MIT: (a) as a Quantum Critical Transition, signaled by an effective gap going to zero; and (b) as a Quantum Percolation Transition, where a sample-spanning metallic percolation path is formed as the fraction of the hopping bonds in the transport paths is going to zero. We found that the Quantum Percolation Transition theory provides a better description of the MIT. We also observed an anomalously low gap region next to the MIT. We discuss the relevance of our results in the light of recent experimental measurements.

## Introduction

Semiconductor nanoparticle (NP) solids have received much attention because their electronic and optical properties are promising for numerous applications^1^, including third generation solar cells^2, 3^, light emitting diodes^4^, and field effect transistors (FET)^5, 6^. Nanoparticle solids promise great control of key device parameters via the Quantum Confinement effect: the energy levels and optical properties of individual NPs can be tuned by adjusting their diameters, and by engineering their surfaces, ligands, core-shell structures, and chemical composition^7, 8^.

NPs hold great promise specifically for solar cell applications, as crucial performance controllers like the absorption onset at the band gap can be optimized by changing the NP diameter. Quantum Confinement can also improve solar cell efficiency by opening new energy conversion channels. Most notable among them is the down-converting Carrier Multiplication (CM) conversion channel, where an absorbed high energy photon generates more than one lower energy electron-hole pairs, also known as Multiple Exciton Generation (MEG)^2, 8–11^. CM/MEG has the theoretical potential to boost the efficiency of solar cells well over the Shockley-Queisser limit^12^. Encouragingly, CM/MEG was already demonstrated in colloidal NPs^13–17^, and also in functioning solar cells^18, 19^.

Very recently, we have advocated that NP solar cells are also an attractive platform to implement a complementary, up-converting energy conversion channel: the Intermediate Band upconversion paradigm^20^.

However, the following Quantum Confinement Dilemma (QCD) poses a substantial challenge for the utilization of NPs. The very same Quantum Confinement that makes NPs conveniently adjustable solar energy absorbers and optoelectronic platforms, also tends to localize the photo-generated charges to the NPs, thereby hindering their transport and eventual extraction from layers of NPs. This dilemma needs to be transcended by tipping the balance towards efficient charge transport while at the same time preserving the quantum confinement of the electronic states. Transcending the Quantum Confinement Dilemma is a major challenge for realizing the promise of NP optoelectronic devices.

Experimentally, transport is often studied in FET setups. Most transport experiments in NP films and FETs reported a thermally activated hopping conductivity, indicating that the NP films are typically in an insulating phase. At very low temperatures, non-interacting variable range hopping^21^, or its interacting, Efros-Shklovskii^22^ counterpart can take place, while at higher temperatures, nearest neighbor activated hopping often dominates^23, 24^. These hopping processes can be further classified into single phonon-assisted Miller-Abrahams hopping, or multi-phonon-assisted Marcus hopping processes.

The value of hopping mobility in these insulating NP solids is typically low, 10^−3^–10^−2^ cm^2^/Vs. Therefore, to establish the utility of NP films for optoelectronic applications, it is imperative to find ways to drive NP films from their insulating phase across a Metal-Insulator Transition (MIT) into a conducting phase. Various groups attempted to boost the mobility by boosting the inter-NP transition rate with a variety of methods, including: ligand engineering^25–27^, band-alignment engineering^28^, chemical-doping^29, 30^, photo-doping^31^, metal-NP substitution^32^, epitaxial attachment of NPs^33, 34^, and atomic layer deposition (ALD) methods^35^.

Encouragingly, these efforts recently translated into progress, as NP films were reported to exhibit band-like, temperature-insensitive mobilities, with values approaching 10 cm^2^/Vs at room temperatures. Interestingly, among these works, the ALD infilled NP solids also showed noticeable CM/MEG energy conversion efficiencies^36, 37^.

Building on the successful boost of charge transport across the MIT, recently, a few groups succeeded forming NP solar cells with overall power conversion efficiencies approaching and even exceeding 10%, a crucial threshold for commercialization^28, 38, 39^.

In spite of the experimental excitement in the field, theoretical modeling of the transport in NP solids did not keep up with the rapid experimental progress. First, an all-out band structure approach to NP solids is not realistic due to the forbiddingly large time-, energy-, and length-scales. The disorder of NPs cannot be captured in a superlattice approach either; and including the ligands is prohibitive even more. Finally, band structure transport calculations beyond the linear response regime would require a complete recalculation of the electronic wavefunctions for every bias voltage, and thus have not been attempted at all.

Therefore, a computable transport model has to follow a multi-scale and hierarchical programme: (a) it has to build a microscopic foundation with an ab initio description of individual NPs on the scale of a few nanometers; (b) followed by modeling inter-NP transitions on the scale of 10 nm; and (c) completed by a description of mesoscopic transport across a disordered array of NPs on the scale of several hundred to a few thousand nanometers. (d) Finally, the theory needs to be able to handle both insulating and metallic transport in NP films, which is very challenging as the natural basis for the former is the set of localized electronic wavefunctions, while for the latter it is the extended electronic states.

Because of the difficulty of these requirements, this programme has not been carried out in general. Important early steps in this direction started with the pioneering study of Chandler and Nelson of charge transport of NP arrays^40^. They modeled the individual NPs by a band structure method, albeit on k · p level. Then they performed a Monte Carlo transport study on extremely small samples of 2 × 2 × 3 and 3 × 3 × 4 NPs. One of their qualitative messages was to show how important are interactions effects, in particular the on-site Coulomb interaction, to understand transport experiments.

In parallel, the organic solar cell community has built a tool-set based on semiclassical rate equations to study charge generation, transport and collection^41–46^. Also, the charge transport properties of silicon nanoparticles embedded in a silicon oxide matrix were explored using a Kinetic Monte Carlo (KMC) method^47, 48^. Although these papers built an advanced method that was capable of handling short and long range Coulomb interactions, the small system sizes did not allow them to draw definitive conclusions.

Our own first contribution was to build on these works by developing a Kinetic Monte Carlo method to compute the electron and hole mobilities as a function of the NP diameter^49^. We found that the mobility exhibited a plateau or maximum as a function of the NP diameter. We showed that this was the result of competition between interaction and kinetic effects. At small NP diameters, the mobility increased with increasing NP diameters because fewer and fewer hops were needed to travel the same distance. At larger diameters, charges faced a Coulomb barrier more often as they attempted to hop on NPs which were already occupied by another charge with a higher probability, and this Coulomb blockade hindered their transport. This finding was in agreement with corresponding experiments^50, 51^.

Electronic transport was also studied using ab initio methods. Some approached the problem from the hopping limit using Marcus theory^52–56^, while an other work explored conductance of Si NP devices using the Landauer formalism^57^. Both approaches underlined the crucial importance of atomistic details of the NP surface. However, neither of them was capable of taking into account size disorder effects, and typically the NPs were small. Yet others focused on model Hamiltonian approaches and built a qualitative understanding of NP FETs^58^, photoconductors^59^ and studied the effect of disorder in NP films providing some initial insights into the mechanism of MIT^60–63^. After all these efforts, a comprehensive transport theory of the MIT in NP solids is still not available and is very much needed.

## Results

The present paper reports an effort to carry out the above-outlined, full programme of developing a hierarchical, multi-scale transport model for NP films. We have developed a framework that captured the physics on the above three levels: (a) from the semi-empirical description of the electronic structure of individual NPs; through (b) the modeling of inter-NP transitions; completed by (c) feeding the results of (a)-(b) into a mesoscale dynamical model of electrons moving across the entire NP layer with a thickness of several hundred nanometers. Our results were analyzed in terms of two possible theoretical frameworks, which we call Quantum Critical and Quantum Percolation transitions. The different structures of these two theories are clearly described, and their places within the large field of Metal-Insulator Transitions are articulated. Eventually, our analysis concluded that our results were most naturally interpreted within the Quantum Percolation framework.

The presentation and discussion of our results requires a brief description of the levels of our hierarchical model. (1) We adapted a k · p calculation of the energy levels of PbSe NPs in a broad diameter range of 3–8 nm, which have been validated via comparison to optical experiments^64^. Our hierarchical model is modular, and in the future these k · p-calculated energy levels can be replaced by full ab initio results. Our model also included the electron-electron interaction on the level of on-site/self-charging energy. This self-charging energy can be calculated by a variety of methods, including the semi-empirical pseudopotential configuration interaction method of Zunger and coworkers^65, 66^ and the tight-binding based many body perturbation theory method of Delerue^67^. In this paper we report results with the latter approach.

(2) On the next length scale of the order of 10 nm, we modelled the hopping transitions between neighboring NPs. We incorporated into our model (2a) Miller-Abrahams single phonon-assisted activated hoppings, and (2b) Marcus-theory based multi-phonon activated transitions. In this paper we only report results with the Miller-Abrahams hopping. The transition rates were calculated by assuming that the NPs are separated by twice the ligand length.

(3) Models with only activated hopping are unable to describe the MIT because transport in the metallic phase is not activated. To be able to reach the MIT, we extended our model with an additional, non-activated metallic transition channel between neighboring NPs whose LUMO energies differed by less than an Overlap Energy, OE. This Ohmic metallic transition channel, parallel to the hopping transition channel, represents that when the Overlap Energy, the perturbation of an NP’s energy levels by the neighboring NP, exceeds the LUMO energy difference of the NPs, then the induced perturbative mixing delocalizes the LUMO of the NP over *both* NPs.

With the introduction of this quantum-delocalization process, our extended model has the potential to reach the MIT when the non-activated, quantum delocalized metallic transitions finally span the entire sample as OE is increased. Obviously, the accuracy of this extended model to describe transport *inside* the metallic phase decreases as the MIT is crossed, as the phases of the extended wavefunctions are not incorporated.

Experimentally, the OE can be tuned by varying the wavefunction overlap by Atomic Layer Deposition (ALD)^35^, ligand engineering^25^, or NP substitutions^32^, among others.

**Figure 1:**
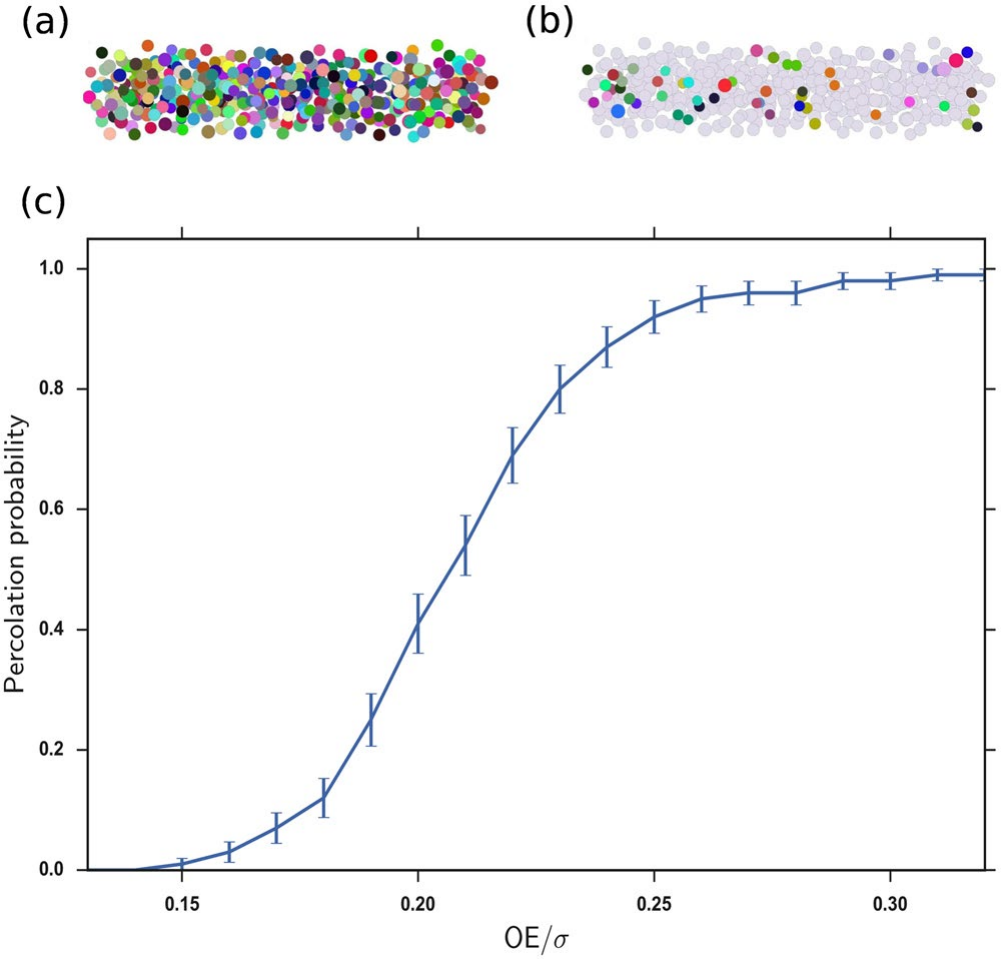
(**a**) A close-packed array of NPs with size disorder; (**b**) An NP array with a sample spanning metallic cluster; (**c**) The percolation probability of sample spanning metallic clusters as a function of *OE*/*σ*.

(4) On the hierarchically top length scale of hundreds to a few thousand nanometers, we generated an entire solid sample of the NPs, developed and pair-wise coupled in steps (1)–(3). We used the event-driven Molecular Dynamics code PackLSD^68^ to obtain close packed (jammed) NP solids. Each sample contained several hundred to a few thousand NPs. To represent randomness, the NP radii were selected from a Gaussian distribution. Figure 1(a) illustrates a typical generated NP solid sample.

(5a) To simulate transport across the entire generated NP solid sample, we identified the pairs of neighbouring NPs whose LUMO energies were closer than the preselected OE and therefore were coupled by non-activated transitions. (5b) Then we performed an advanced quick-union algorithm to identify “metallic clusters”, formed by NPs coupled by non-activated transitions across the entire cluster. Figure 1(b) illustrates an NP sample where the metallic cluster spans the entire sample.

(6) Next, we injected electrons into the NP solid to reach a predetermined electrons/NP density.

(7) Finally, we developed an Extended Kinetic Monte Carlo (KMC) code that incorporated activated transitions^49, 69^ between all neighboring NPs, and the additional non-activated transitions between neighboring NPs with a site energy difference less than OE, to simulate the transport of electrons across the entire NP solid. Our central quantity of interest was the electron mobility. We always made sure that the voltage was sufficiently small to keep our simulations in the linear I-V regime. Doing so included the observation that in each sample, the disorder of the NP energies did not exactly average to zero, and thus generated an internal bias field. We eliminated this bias by always taking the pairwise average of the currents with a forward and a backward applied voltage.

We selected the typical ligand lengths, the NP-NP separation, and the overall hopping attempt rate prefactor such that our mobilities are consistent with the experimental values of the Law group^50^. It is noted that the overall magnitude of the mobility is exponentially sensitive to these factors.

We systematically explored wide parameter regions, including that of temperature, disorder, electron density and Overlap Energies. For each parameter set, we simulated at least 40, typically several hundred samples.

To characterize the energy disorder induced by the NP size disorder, we plotted the histogram of the NP LUMO energies and extracted its FWHM, *σ*. In our model, the MIT is driven by the competition of the disorder of LUMO energies with this width of *σ* that tends to localize the electrons to individual NPs, against quantum delocalization, driven by the overlap energy OE of electronic states of neighboring NPs. Therefore, the dimensionless ratio (*OE*/*σ*) is a natural control parameter for tuning across the MIT. We simulated our model over a wide range of the (*OE*/*σ*) control parameter, and report our results next.

First, since the competition of the localizing and delocalizing tendencies plays out over a percolation transition, we started our analysis by characterizing the formation of sample-spanning, percolating metallic clusters. Figure 1(c) illustrates the probability of the emergence of a sample-spanning percolating cluster as a function of (*OE*/*σ*) in our samples with 400 NPs Fig. 1(c) shows a broadened percolation transition with a center around (*OE*/*σ*)_*p*,*c*_ ≈ 022, and with an onset of (*OE*/*σ*)_*p*,*o*_ ≈ 0.13.

We explored the size dependence of this transition by simulating systems with 800 NPs. As described in the Supplementary Information, we found that the onset of the percolation transition (*OE*/*σ*)_*p*,*o*_ showed a limited size dependence by shifting to ≈0.125, while the width of the percolation transition narrowed.

In each sample, one expects the MIT to occur for (*OE*/*σ*) ≈ (*OE*/*σ*)_*p*,*c*_ of that particular sample, because for (*OE*/*σ*) < (*OE*/*σ*)_*p*,*c*_ the transport path unavoidably involves thermally activated hops, while for (*OE*/*σ*) > (*OE*/*σ*)_*p*,*c*_ the percolating metallic cluster shunts the activated transitions. The critical percolation points (*OE*/*σ*)_*p*,*c*_ vary from sample to sample. This variation is responsible for the broadening of the averaged percolating transition in Fig. 1(c). The precise location of the MIT with respect to the onset of percolation will be discussed below.

**Figure 2:**
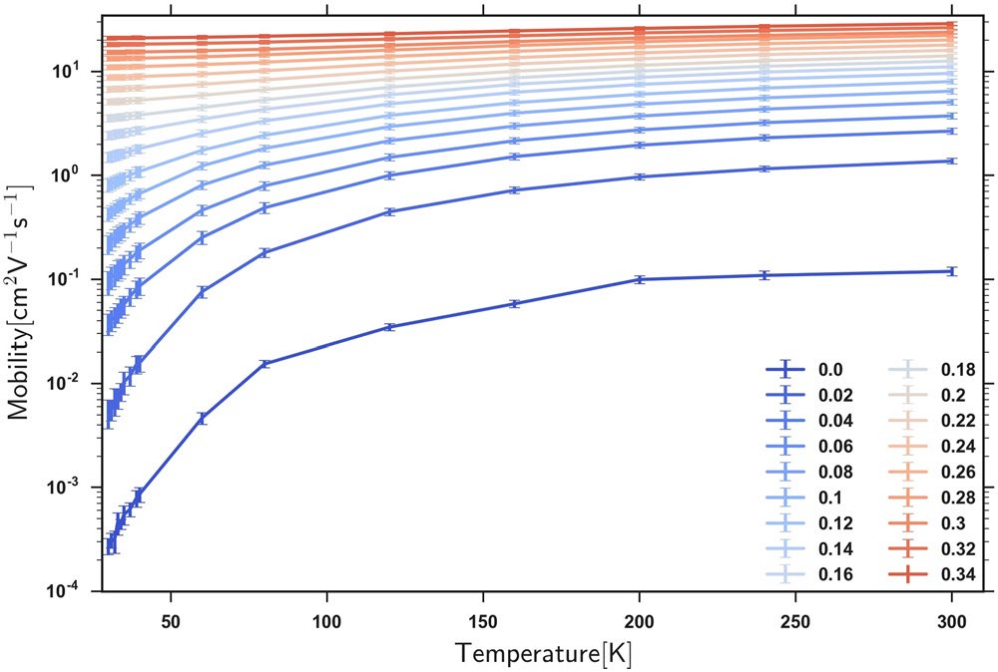
The temperature dependence of the electron mobility for a range of (*OE*/*σ*) sweeping from 0 to 0.34.

Figure 2 illustrates the electron mobility as a function of temperature, as (*OE*/*σ*) is varied across the percolation transition of Fig. 1(c), while keeping the electron density fixed. Visibly, the mobility data exhibit an MIT: at low overlap energies OE, the transport occurs by thermally activated hopping, whereas at high overlap energies OE the transport transforms into a temperature-independent metallic behavior.

We now proceed to the analysis of our results. One of the widely used frameworks to analyze models of MIT is the Quantum Critical Theory (QCT). In QCT, the model’s ground state exhibits a phase transition as one of the quantum parameters is tuned^70, 71^. In the case of the MIT, the natural quantum control parameter is the ratio of the disorder to the energy scale that drives the metallic behavior. In our model, this quantum control parameter is the aforementioned *OE*/*σ*. The phase boundary to the insulating phase is best identified from the vanishing of the gap of the model’s excitation spectrum. A natural way to determine this gap is to fit the temperature dependence of the mobility with a thermally activated form at the lowest accessible temperatures. (While this method involves studying finite temperature data, these *T* > 0 data are taken at the lowest accessible temperatures and are only used to extract the *T* = 0 gap. As such, this protocol is an appropriate method to analyze a *T* = 0 quantum phase transition).

**Figure 3:**
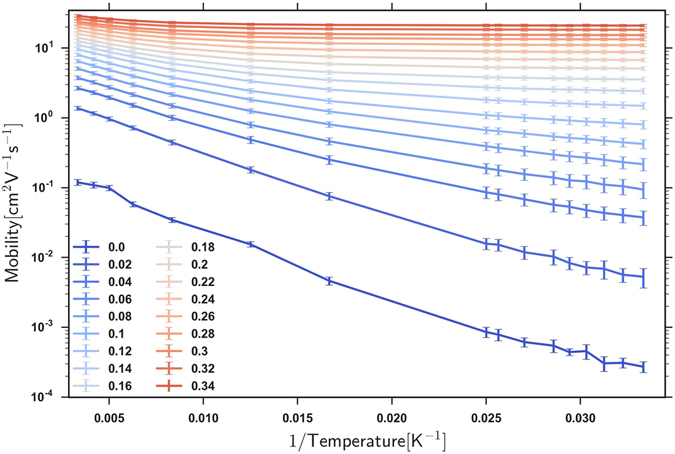
The logarithm of the mobility as a function of 1/T, for the determination of an effective gap *T*_0_, for the same range of (*OE*/*σ*) as in Fig. 2.

We plotted the log(mobility) as a function of 1/T in Fig. 3, and identified an effective activation gap *T*_0_ from the slope of this log(mobility) vs. 1/T graph. We performed the fit at the lowest temperatures so that the *T*_0_ activation gap is closest to the *T* = 0 quantum gap. At higher temperatures, the effective gap increases for several reasons, including that the electrons are exploring higher gap transport paths.

**Figure 4:**
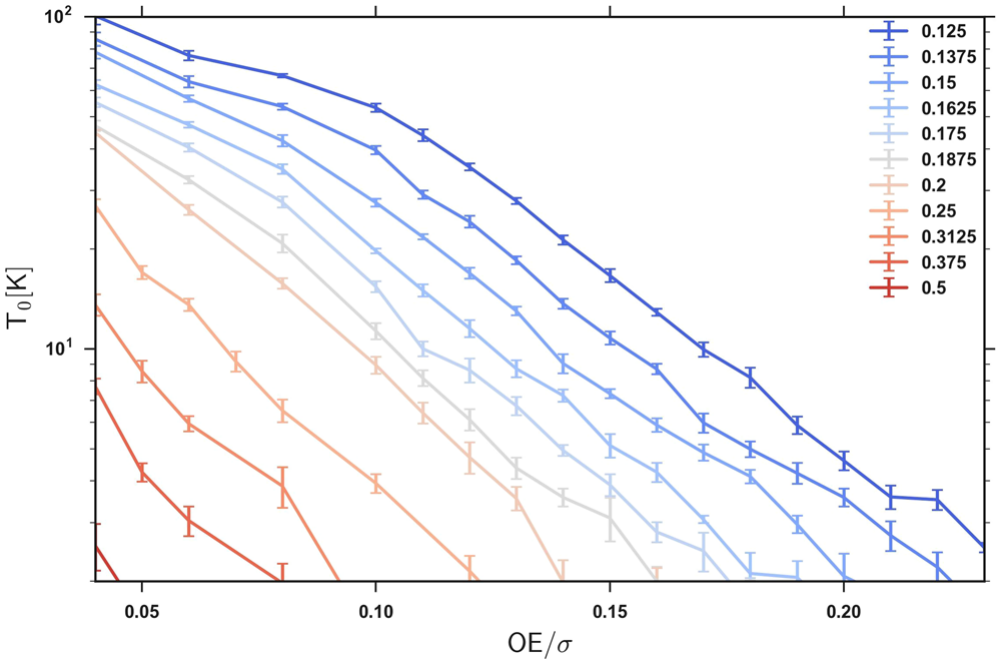
The effective gap *T*_0_ as a function of (*OE*/*σ*) for electron densities varying from 0.125 to 0.5 electron/NP.

The same procedure was carried out for a range of electron densities. Figure 4 shows the determined activation gap *T*_0_ for the studied range of electron densities. Visibly, with increasing *OE*/*σ*, *T*_0_ decreases towards zero, indicating the onset of the metallic transport.

However, Fig. 4 shows a surprise: for a range of electron densities *T*_0_ approaches zero at (*OE*/*σ*) values well below (*OE*/*σ*)_*p*,*c*_ ≈ 0.22 and even below (*OE*/*σ*)_*p*,*o*_ ≈ 0.13. Given that the metallic bonds do not percolate in such samples, the appearance of gapless transport within this QCT analysis is at odds with the natural expectations.

This surprising discrepancy can be partially due to the finiteness of the simulated samples. As discussed in relation to Fig. 1(c), in half of the samples the percolation transition occurs at values of the control parameter (*OE*/*σ*) smaller than the averaged critical value (*OE*/*σ*)_*p*,*c*_ ≈ 0.22. This broadens the percolation transition and explains the lower onset of the percolation probability at (*OE*/*σ*)_*p*,*o*_ ≈ 0.13 in Fig. 1(c). At a given *OE*/*σ*, the ensemble average mobility can be dominated by the limited number of percolating samples with their high mobilities, making the average *T*_0_ seemingly vanish.

However, the broadening of the transition can account for the vanishing of *T*_0_ only above (*OE*/*σ*_*p*,*o*_) ≈ 0.13, whereas Fig. 4 shows that for a range of densities *T*_0_ vanishes for (*OE*/*σ*) values well below (*OE*/*σ*_*p*,*o*_) ≈ 0.13. This suggests that it remains a challenge to explain our results within the QCT theory.

In hindsight, this was not surprising since percolative aspects of the physics are not built into the standard QCT theories, whereas they are crucial in our model. Therefore, it is worth exploring, whether alternative physical pictures can provide a better description of the MIT in our system.

Motivated by this, we turned to the early work of Efros and Shklovskii (ES), who considered a network of bonds, some of them having high conductivity, the others low but non-vanishing conductivity^72^. This ES model is an extension of the widely-cited percolative resistor network model^73, 74^, where the bonds of the network are either conducting, or not at all.

The ES network had “dielectric” (low conductivity) and metallic, high conductivity bonds, and therefore had a critical crossover from a low conductivity region to a high conductivity region when the high conductivity bonds percolated. However, ES did not consider the temperature dependence of the conductivity of these bonds, and thus even at *T* = 0 did not have a true MIT. Moreover, they did not develop an analysis of the overall network conductivity based on the temperature dependence of the mobility.

Thus, the step forward in our paper is that we are not studying only a critical crossover from a low conductivity regime to a high conductivity regime. As a qualitative progress, our “extended ES percolation model” links the percolation transition of high conductivity bonds to the qualitative change in the temperature dependence of the system mobility from activated to metallic, thus defining a new class of the MIT. We call this MIT class the Quantum Percolation Transition, QPT.

Before proceeding, we articulate the position of our extended ES percolation model within the large field of MIT transitions. The theoretical investigations of the MIT started with Anderson’s 1958 paper^75^, followed by a long list of notable contributions^76–81^. Within the vast field of MIT, the class where our model belongs can be articulated as follows. (a) Most of the early papers studied non-interacting electrons, whereas the electrons in our simulations are interacting, with an on-site repulsion. (b) Most of the papers approach the MIT from the metallic side, whereas we approach the MIT from the insulating side. (c) Most theories are formulated using the electronic wavefunctions, and often derive the MIT from tracking the interference of the phases of these wavefunctions. In contrast, our method is not suited for tracking the phase of the wavefunctions. (d) We know of very few theories that approach the MIT from the insulating side. The notable ones among them, e.g. the locator expansion of Anderson^77^, are again theories of non-interacting electrons, where the metallic phase emerges from tracking the wavefunction. As before, we are not studying the wavefunction, further, our electrons are interacting. (e) Most MIT papers study bulk disordered systems, while we have studied nanoparticle solids with their own peculiarities. (f) Most MIT papers do not refer to percolation ideas, whereas percolation is central for our analysis. (g) Hopping conductivity models can approach the MIT but cannot reach it, since all transitions are activated. In contrast, our model adopted an additional, metallic, non-activated transition channel, which enables us to actually reach the MIT. Having articulated these differences relative to the majority of the MIT literature, our approach has the potential to deliver and characterize a unique MIT scenario.

Returning to the exposition of our model, Efros and Shklovskii developed a scaling theory of the conductivity of a system where nodes were connected by either low conductivity bonds *σ*_*low*_, or high conductivity bonds *σ*_*high*_. They proposed that a universal, critical crossover scaling function describes the system mobility as the increasing fraction of high mobility bonds percolate across the system:

1 $$\sigma /{\sigma }_{high}={({\sigma }_{low}/{\sigma }_{high})}^{s}\varphi [({x}_{c}-x)/({\sigma }_{low}/{\sigma }_{high}{)}^{m}]$$

wherein *ϕ*(*z*) is a universal analytic scaling function, and (*x*_*c*_ − *x*) measures the distance of the control parameter *x* from its critical value *x*_*c*_. Finally, *s* and *m* are universal critical exponents. *s* = 1 in 2 dimensions, and *s* = 1/2 in the effective medium, or mean field-theory. Their work did not determine the specific form of the universal scaling function *ϕ*(*z*), only its natural scaling variable, *z* = (*x*_*c*_ − *x*)/(*σ*_*low*_/*σ*_*high*_)^*m*^.

Based on general principles, *ϕ*(*z*) is an analytic function around the critical point with *ϕ*(0) = 1. On physical grounds we adopt the approximation *ϕ*(*z*) = 1/(1 + *az*) for the scaling function, which satisfies these two constraints. Here *a* is a parameter. This choice is motivated by an “effective medium” theory of our “two conducting channels” model, where we approximate every bond as an “effective bond”, comprising a *σ*_*low*_ conductivity component with a weight of (*x*_*c*_ − *x*) and a *σ*_*high*_ conductivity component with the remaining weight in the controlling conduction path, and envision the two components being electrically coupled in series. Coupling the components in series ensures that if only a small fraction of the low conductivity, insulating bonds are left in the transport path, they still drive the overall conductivity insulating at low temperatures, as they should, since the metallic bonds have not yet percolated across the entire sample. Integrating our approximation of the scaling function into the Efros-Shklovskii theory, we have analyzed our data with the overall Quantum Percolation Transition QPT form:

2 $$\sigma (x,T)={\sigma }_{metal}{({\sigma }_{hop}\exp (-{T}_{0}/T)/{\sigma }_{metal})}^{s}[\mathrm{1/}[1+a({x}_{c}-x)/({\sigma }_{hop}\exp (-{T}_{0}/T)/{\sigma }_{metal}{)}^{m}]]$$

where *σ*_*hop*_exp(−*T*_0_/*T*) is the activated conductivity of the hopping component of the effective bond, whereas *σ*_*metal*_ is the non-activated metallic conductivity of the metallic component. The “series coupling of the conductivity components” interpretation is most appropriate for *m* = 1. We therefore also used *m* = 1 and the corresponding *s* = 1^72^. In this picture, the physics of the MIT is that (*x*_*c*_ − *x*), the fraction of the activated bonds in the path that controls the transport goes to zero as we approach the MIT from the insulating phase. We identified *x* with our natural control parameter (*OE*/*σ*). We determined the location of the MIT phase boundary by fitting our results with the above QPT scaling form, using *x*_*c*_ − *x* = (*OE*/*σ*)_*MIT*_ − (*OE*/*σ*) and extracting *x*_*c*_ = (*OE*/*σ*)_*MIT*_. We note that while the above theories were formulated for the conductivity, the scaling form of the mobilities is the same.

There are stark formal and conceptual differences between the QCT and the QPT theories. In the QCT theory, the MIT is driven by the transport gap going critical. Formally, this manifests itself via an exponential criticality: the gap in the activation exponential of the system mobility is going critical. In contrast, in the QPT theory, the MIT is driven by the fraction of the hopping conductivity bonds in the overall transport path going critical. Formally, this manifests itself by the pre-exponential factor of the activated conductivity component of the system mobility going critical, while the gap of the individual hopping bonds remaining finite across the MIT.

**Figure 5:**
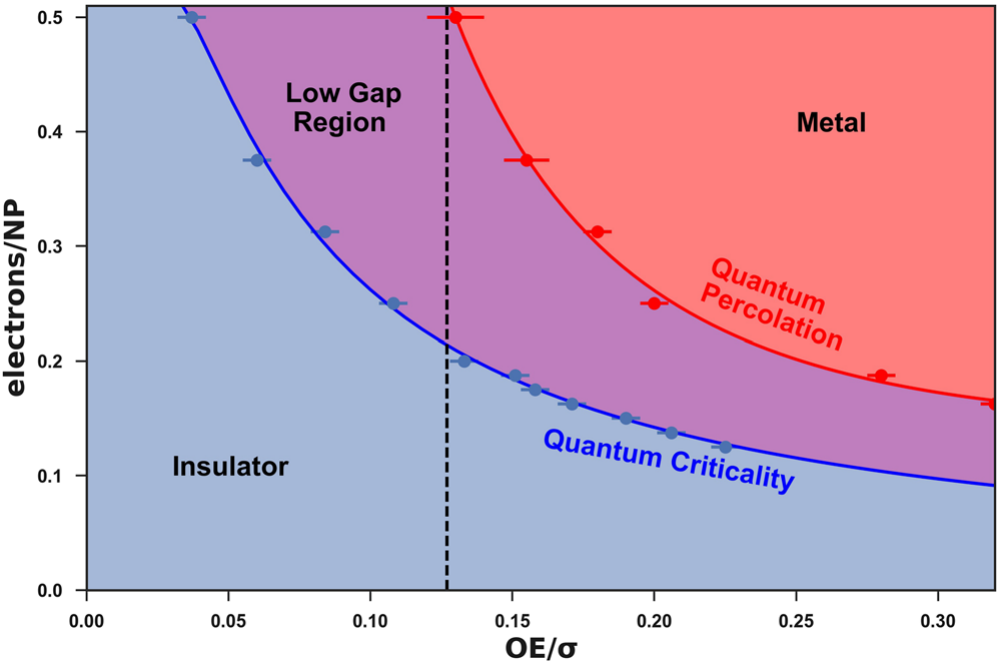
Phase diagram on the electron density vs. (*OE*/*σ*) plane.

Figure 5 shows the phase diagram on the electron density vs. (*OE*/*σ*) plane, determined by analyzing the data in the QPT framework. The MIT is indicated by the red phase boundary. For comparison, we also show the “phase boundary” that emerges from the QCT framework.

To analyze this phase diagram, first, we refer back to the earlier discussion that when an MIT phase boundary was determined via the exponentially critical QCT theory (blue line), it visibly extended to (*OE*/*σ*) values well below even the onset of the percolative transition (*OE*/*σ*)(*p*,*o*). As discussed, we were unable to develop a credible explanation for such a behavior. In contrast, when we later determined an MIT phase boundary via the pre-exponentially critical QPT theory (red line), the MIT transition occurred at considerably higher (*OE*/*σ*)_*MIT*_ values. The (*OE*/*σ*)_*MIT*_ values remained above the onset of the percolation transition (*OE*/*σ*)_*p*,*o*_ ≈ 0.13 for all electron densities. Since this latter result is physically acceptable, indeed natural, we conclude that the Quantum Percolation Transition framework gives the physically credible framework for our results, instead of a Quantum Critical Theory.

Even after adopting the QPT framework, a remnant of the QCT “phase transition” persists in the form of an anomalously low gap region next to the QPT MIT. This is because if the mobility data were analyzed in the insulating regime in terms of the one component, purely gap-activated QCT form instead of the two-component QPT form, then the QCT gap of the mobility between the blue and red lines will appear vanishingly small, as the fractional contribution of the gapped component of the system mobility is going to zero as the MIT is approached.

We tested this physical picture as follows. In our simulations, we tracked the energy-differences of every individual executed electron transition as the MIT was approached. The distribution of these energy differences was bi-modal: many of the executed transitions were non-activated, “zero-energy” transitions either because of the energy difference was negative in our voltage-biased transport simulation, or it was positive but less than *OE*. There was a distinct second peak of the distribution, at “gaps” larger than *OE* that did not exhibit any critical softening, i.e. the energy differences did not shift toward lower values on approaching the MIT. This observation confirmed that the apparent softening of the QCT mobility gap was not caused by the inherent gap going critical, but by the fraction of the executed, gapped transitions going to zero, relative to the fraction of the non-gapped transitions. This observation is a further evidence for the QPT being the appropriate theory of our simulations.

Regarding the experimental comparisons, our simulations should be quite directly applicable to the work of the Law group, who changed the Overlap Energy between their PbSe nanoparticles by the Atomic Layer Deposition (ALD) of alumina in a FET setup of their NP layer^35^. They managed to increase the mobility by this ALD process with two orders of magnitude, to *μ* ≈ 7 *cm* ^2^/*Vs*. Their mobility evolved from a hopping behavior to a temperature independent, metallic behavior. This suggests that the NP films were driven to the MIT, and possibly across it. Until now, however, data was made available on only a few samples, so that a quantitative analysis in terms of our QPT theory has to wait until more samples in the critical regime of the MIT are measured.

Another exciting work in this realm was carried out by the Murray-Kagan group^32^. They tuned the mobility of a CdSe NP film by substituting Au or Ag NPs for a fraction of the CdSe NPs of the host matrix. The mobility *μ* of the system increased by about four orders of magnitude over a narrow range of Au NP concentration at a fixed temperature. They qualitatively analyzed their data in terms of a percolative picture. While our simulations were not set up to precisely model substitutionally disordered systems, our model shares the same basic paradigm: the MIT in both systems is driven by the formation of sample-spanning quantum percolating metallic clusters. As more data becomes available concerning their work, a quantitative analysis should be carried out regarding the applicability of the QPT theory of their substitutionally disordered samples.

Interesting experimental work was also carried out on the percolative aspects of transport in porous Si^82–84^. It would be interesting to comparatively re-analyze these original data in terms of the competing QCT and QPT theories.

We close this discussion with a speculative remark. As discussed above, NP solids and layers will be regarded as serious contenders for solar and optoelectronic applications when the charge mobility crosses the MIT and gets well within the metallic phase. A key limiting factor of the mobility is that it is particularly hard to ensure the uniformity of the NP sizes. The NP size disorder translates into a substantially disordered energy landscape that hinders transport.

Our simulations suggest that the transport in NP solids may be dominantly percolative. In this physical picture, the electrons mostly flow through only the percolating clusters, where the NP energies are much more finely matched, and therefore the electrons experience a much smaller effective disorder during transport than the nominal disorder. This insight gives hope that the effective disorder in percolative systems is substantially smaller than in homogeneously disordered systems. This suggests that the limits on the transport in NP solids may be more easily overcome than thought before, and the utilization of NP solids for electronic applications may be more attainable, than previously thought.

## Discussion

In this paper we reported the development of a multi-scale, extended Kinetic Monte Carlo (KMC) model of transport in nanoparticle systems. We extended “hopping-only” KMC methods by additionally enabling a quantum delocalization transport channel via non-activated transitions between NPs. This extension made it possible for the first time to simulate transport in disordered, interacting NP systems all the way to the MIT. We presented two theoretical frameworks to analyze our simulation results. In a Quantum Critical Theory (QCT), the effective transport gap is expected to go to zero at the MIT, and therefore the MIT is characterized by an exponential critical behavior. In contrast, in the Quantum Percolation Theory (QPT), the MIT is driven by the fraction of hopping bonds in the path that controls the transport going to zero. As such, the QPT exhibits a distinctly different, pre-exponential criticality. Our analysis of the results of the extended KMC simulations suggested that the Quantum Percolation Theory provided a more credible description of the MIT and the phase diagram. We closed by analyzing recent experiments on NP systems in FET geometries that reported reaching and crossing the MIT. It will be exciting to see whether the QCT or the QPT will provide a better description of future, more detailed experiments in nanoparticle systems. Possibly, each of these theories will be applicable for classes of experimental systems. We found that in our NP system, transport in NP solids had a strong percolative character that experiences an effective disorder smaller than the nominal disorder. This gives hope that NP solids can be pushed to, and well beyond the Metal Insulator Transition, towards successful utilization for optoelectronic and photovoltaic applications in the near future.

## Methods

We have developed a hierarchical, multi-level Kinetic Monte Carlo theory of transport in nanoparticle systems. The seven main levels of our method were described very briefly in the main text in order to provide context for the presentation of the results and their discussion. The details of our methods are provided in the Supporting Information, available online. The datasets generated during and analysed during the current study are available from the corresponding author on reasonable request.

## Electronic supplementary material

Supplementary Information

## References

[1] Talapin DV, Lee J-S, Kovalenko MV, Shevchenko EV (2010). Prospects of colloidal nanocrystals for electronic and optoelectronic applications. Chemical Reviews.

[2] Nozik AJ (2002). Quantum dot solar cells. Physica E: Low-dimensional Systems and Nanostructures.

[3] Kamat PV (2008). Quantum dot solar cells. Semiconductor nanocrystals as light harvesters. The Journal of Physical Chemistry C.

[4] Shirasaki Y, Supran GJ, Bawendi MG, Bulović V (2013). Emergence of colloidal quantum-dot light-emitting technologies. Nature Photonics.

[5] Talapin DV, Murray CB (2005). PbSe nanocrystal solids for n- and p-channel thin film field-effect transistors. Science.

[6] Hetsch F, Zhao N, Kershaw SV, Rogach AL (2013). Quantum dot field effect transistors. Materials Today.

[7] Kovalenko MV (2015). Prospects of nanoscience with nanocrystals. ACS Nano.

[8] Wippermann S (2013). High-pressure core structures of si nanoparticles for solar energy conversion. Physical Review Letters.

[9] Vörös M, Rocca D, Galli G, Zimanyi GT, Gali A (2013). Increasing impact ionization rates in si nanoparticles through surface engineering: A density functional study. Physical Review B.

[10] Vörös M (2014). Germanium nanoparticles with non-diamond core structures for solar energy conversion. Journal of Materials Chemistry A.

[11] Govoni M, Marri I, Ossicini S (2012). Carrier multiplication between interacting nanocrystals for fostering silicon-based photovoltaics. Nature Photonics.

[12] Shockley W, Queisser HJ (1961). Detailed balance limit of efficiency of p-n junction solar cells. Journal of Applied Physics.

[13] Klimov VI (2014). Multicarrier interactions in semiconductor nanocrystals in relation to the phenomena of auger recombination and carrier multiplication. Annual Review of Condensed Matter Physics.

[14] Nair G, Chang L-Y, Geyer SM, Bawendi MG (2011). Perspective on the prospects of a carrier multiplication nanocrystal solar cell. Nano Letters.

[15] Beard MC, Luther JM, Semonin OE, Nozik AJ (2013). Third generation photovoltaics based on multiple exciton generation in quantum confined semiconductors. Accounts of Chemical Research.

[16] Padilha LA (2013). Carrier multiplication in semiconductor nanocrystals: Influence of size, shape, and composition. Accounts of Chemical Research.

[17] Shabaev A, Hellberg CS, Efros AL (2013). Efficiency of multiexciton generation in colloidal nanostructures. Accounts of Chemical Research.

[18] Semonin OE (2011). Peak external photocurrent quantum efficiency exceeding 100% via meg in a quantum dot solar cell. Science.

[19] Zhai G (2012). Quantum dot PbS(0.9)Se(0.1)/TiO2 heterojunction solar cells. Nanotechnology.

[20] Vörös M, Galli G, Zimanyi GT (2015). Colloidal nanoparticles for intermediate band solar cells. ACS Nano.

[21] Guyot-Sionnest P (2012). Electrical transport in colloidal quantum dot films. The Journal of Physical Chemistry Letters.

[22] Efros A, Shklovskii B (1975). Coulomb gap and low-temperature conductivity of disordered systems. J. Phys. C-Solid State Phys..

[23] Yu D, Wang C, Wehrenberg BL, Guyot-Sionnest P (2004). Variable range hopping conduction in semiconductor nanocrystal solids. Physical Review Letters.

[24] Liu H, Pourret A, Guyot-Sionnest P (2010). Mott and efros-shklovskii variable range hopping in CdSe quantum dots films. ACS Nano.

[25] Wang R (2016). Colloidal quantum dot ligand engineering for high performance solar cells. Energy & Environmental Science.

[26] Jang J, Liu W, Son JS, Talapin DV (2014). Temperature-dependent hall and field-effect mobility in strongly coupled all-inorganic nanocrystal arrays. Nano Letters.

[27] Lee J-S, Kovalenko MV, Huang J, Chung DS, Talapin DV (2011). Band-like transport, high electron mobility and high photoconductivity in all-inorganic nanocrystal arrays. Nature Nanotechnology.

[28] Chuang C-HM, Brown PR, Bulović V, Bawendi MG (2014). Improved performance and stability in quantum dot solar cells through band alignment engineering. Nature Materials.

[29] Chen T (2016). Metal-insulator transition in films of doped semiconductor nanocrystals. Nature Materials.

[30] Choi J-H (2012). Bandlike transport in strongly coupled and doped quantum dot solids: A route to high-performance thin-film electronics. Nano Letters.

[31] Talgorn E (2011). Unity quantum yield of photogenerated charges and band-like transport in quantum-dot solids. Nature nanotechnology.

[32] Cargnello M (2015). Substitutional doping in nanocrystal superlattices. Nature.

[33] Savitzky BH (2016). Propagation of structural disorder in epitaxially connected quantum dot solids from atomic to micron scale. Nano Letters.

[34] Whitham K (2016). Charge transport and localization in atomically coherent quantum dot solids. Nature Materials.

[35] Liu Y (2013). PbSe quantum dot field-effect transistors with air-stable electron mobilities above 7 cm^2^ v^−1^ s^−1^. Nano Letters.

[36] Sandeep CSS (2013). High charge-carrier mobility enables exploitation of carrier multiplication in quantum-dot films. Nature Communications.

[37] ten Cate S (2013). Activating carrier multiplication in PbSe quantum dot solids by infilling with atomic layer deposition. The Journal of Physical Chemistry Letters.

[38] Lan X (2016). 10.6% certified colloidal quantum dot solar cells via solvent-polarity-engineered halide passivation. Nano Letters.

[39] Zhitomirsky D (2014). Engineering colloidal quantum dot solids within and beyond the mobility-invariant regime. Nature Communications.

[40] Chandler RE, Houtepen AJ, Nelson J, Vanmaekelbergh D (2007). Electron transport in quantum dot solids: Monte carlo simulations of the effects of shell filling, coulomb repulsions, and site disorder. Physical Review B.

[41] Ries B, Bässler H (1987). Monte carlo study of dispersive charge-carrier transport in spatially random systems with and without energetic disorder. Physical Review B.

[42] Bässler H (1993). Charge transport in disordered organic photoconductors a monte carlo simulation study. physica status solidi (b).

[43] Nelson J, Chandler RE (2004). Random walk models of charge transfer and transport in dye sensitized systems. Coordination Chemistry Reviews.

[44] Groves C (2013). Developing understanding of organic photovoltaic devices: kinetic monte carlo models of geminate and non-geminate recombination, charge transport and charge extraction. Energy & Environmental Science.

[45] Pelzer KM, Darling SB (2016). Charge generation in organic photovoltaics: A review of theory and computation. Molecular Systems Design & Engineering.

[46] Goldey MB, Reid D, de Pablo J, Galli G (2016). Planarity and multiple components promote organic photovoltaic efficiency by improving electronic transport. Phys. Chem. Chem. Phys..

[47] Lepage H, Kaminski-Cachopo A, Poncet A, le Carval G (2012). Simulation of electronic transport in silicon nanocrystal solids. The Journal of Physical Chemistry C.

[48] Lepage, H. *Modélisation de solides à nanocristaux de silicium*. Ph.D. thesis, institut national des sciences appliquées de Lyon (2013).

[49] Carbone I, Carter SA, Zimanyi GT (2013). Monte carlo modeling of transport in PbSe nanocrystal films. Journal of Applied Physics.

[50] Lee J, Choi O, Sim E (2012). Nonmonotonic size-dependent carrier mobility in PbSe nanocrystal arrays. The Journal of Physical Chemistry Letters.

[51] Scheele M (2013). Nonmonotonic size dependence in the hole mobility of methoxide-stabilized pbse quantum dot solids. ACS Nano.

[52] Kaushik AP, Lukose B, Clancy P (2014). The role of shape on electronic structure and charge transport in faceted pbse nanocrystals. ACS Nano.

[53] Chu I-H, Radulaski M, Vukmirovic N, Cheng H-P, Wang L-W (2011). Charge transport in a quantum dot supercrystal. The Journal of Physical Chemistry C.

[54] Vörös M, Brawand NP, Galli G (2017). Hydrogen treatment as a detergent of electronic trap states in lead chalcogenide nanoparticles. Chemistry of Materials.

[55] Brawand NP, Goldey MB, Vörös M, Galli G (2017). Defect states and charge transport in quantum dot solids. Chemistry of Materials.

[56] Goldey, M. B., Brawand, N. P., Vörös, M. & Galli, G. Charge Transport in Nanostructured Materials: Implementation and Verification of Constrained Density Functional Theory. *Journal of Chemical Theory and Computation* **13**, 2581–2590 (2017). 10.1021/acs.jctc.7b00088 28426221

[57] Garcia-Castello N (2013). Silicon quantum dots embedded in a SiO2 matrix: From structural study to carrier transport properties. Physical Review B.

[58] Reich KV, Chen T, Shklovskii BI (2014). Theory of a field-effect transistor based on a semiconductor nanocrystal array. Phys. Rev. B.

[59] Shabaev A, Efros AL, Efros AL (2013). Dark and photo-conductivity in ordered array of nanocrystals. Nano Letters.

[60] Yang J, Wise FW (2015). Effects of disorder on electronic properties of nanocrystal assemblies. The Journal of Physical Chemistry C.

[61] Beverly KC (2002). Quantum dot artificial solids: Understanding the static and dynamic role of size and packing disorder. Proceedings of the National Academy of Sciences.

[62] Remacle F, Levine RD (2000). Electronic response of assemblies of designer atoms: The metal-insulator transition and the role of disorder. Journal of the American Chemical Society.

[63] Fu H, Reich KV, Shklovskii BI (2016). Hopping conductivity and insulator-metal transition in films of touching semiconductor nanocrystals. Phys. Rev. B.

[64] Kang I, Wise FW (1997). Electronic structure and optical properties of PbS and PbSe quantum dots. Journal of the Optical Society of America B.

[65] Wang L-W, Zunger A (1994). Dielectric constants of silicon quantum dots. Phys. Rev. Lett..

[66] An JM, Franceschetti A, Zunger A (2007). Electron and hole addition energies in pbse quantum dots. Phys. Rev. B.

[67] Delerue C, Lannoo M, Allan G (2000). Excitonic and quasiparticle gaps in si nanocrystals. Phys. Rev. Lett..

[68] Donev A, Torquato S, Stillinger FH (2005). Neighbor list collision-driven molecular dynamics simulation for nonspherical hard particles. i. algorithmic details. Journal of Computational Physics.

[69] Bortz A, Kalos M, Lebowitz J (1975). A new algorithm for monte carlo simulation of ising spin systems. Journal of Computational Physics.

[70] Sondhi S, Girvin S, Carini J, Shahar D (1997). Continuous quantum phase transitions. Reviews of modern physics.

[71] Sachdev, S. *Quantum Phase Transitions* (Cambridge University Press, 2013).

[72] Efros AL, Shklovskii BI (1976). Critical behaviour of conductivity and dielectric constant near the metal-non-metal transition threshold. physica status solidi (b).

[73] Broadbent, S. R. & Hammersley, J. M. Percolation processes: I. crystals and mazes. In *Mathematical Proceedings of the Cambridge Philosophical Society*, vol. 53, 629–641 (Cambridge University Press, 1957).

[74] Straley JP (1977). Critical exponents for the conductivity of random resistor lattices. Physical Review B.

[75] Anderson PW (1958). Absence of diffusion in certain random lattices. Physical review.

[76] Mott, N. F. & Davis, E. A. *Electronic processes in non*-*crystalline materials* (OUP Oxford, 2012).

[77] Abou-Chacra R, Thouless D, Anderson P (1973). A selfconsistent theory of localization. Journal of Physics C: Solid State Physics.

[78] Abrahams E, Anderson P, Licciardello D, Ramakrishnan T (1979). Scaling theory of localization: Absence of quantum diffusion in two dimensions. Physical Review Letters.

[79] Finkel’Stein A (1984). Weak localization and coulomb interaction in disordered systems. Zeitschrift für Physik B Condensed Matter.

[80] Altshuler BL, Aronov AG, Lee P (1980). Interaction effects in disordered fermi systems in two dimensions. Physical Review Letters.

[81] Lee PA, Ramakrishnan T (1985). Disordered electronic systems. Reviews of Modern Physics.

[82] Mikrajuddin, Shi F, Okuyama K (2000). Electrical conduction in porous silicon: temperature dependence. Microelectronics Journal.

[83] Ben-Chorin M, Möller F, Koch F, Schirmacher W, Eberhard M (1995). Hopping transport on a fractal: ac conductivity of porous silicon. Phys. Rev. B.

[84] Lampin E, Delerue C, Lannoo M, Allan G (1998). Frequency-dependent hopping conductivity between silicon nanocrystallites: Application to porous silicon. Phys. Rev. B.

