# Supplementary material for: Metal-Insulator Transition in Nanoparticle Solids: Insights from Kinetic Monte Carlo Simulations
Source: Sci Rep. 2017 Aug 1;7:7071. doi: 10.1038/s41598-017-06497-1 (PMC5539282; doi:10.1038/s41598-017-06497-1)
Supplement: Supplementary file 1 — Supplementary Information [file 41598_2017_6497_MOESM1_ESM.pdf]

Supplementary Information for "Metal-Insulator Transition in Nanoparticle Solids: Insights from Kinetic Monte Carlo Simulations"

Luman Qu,† Márton Vörös,‡ and Gergely T. Zimanyi*,†

†*Department of Physics, University of California, Davis*

‡*Materials Science Division, Argonne National Laboratory, Lemont IL 60439*

E-mail: zimanyi@physics.ucdavis.edu

We developed a KMC method to calculate mobilities of size- and lattice-disordered NP arrays. The semi-classical KMC consists of tabulating possible events and then selecting and executing events using a MC-like procedure. In particular, we decided to choose the BKL algorithm.[1] In the BKL method, each time step requires drawing two uniformly distributed random numbers between 0 and 1: r_1 and r_2.

The simulation is initialized and then the time-evolution starts by determining the rates Γ of possible events and then in each step we find the event j for which the below equation is satisfied.

$$\sum_{i=1}^{j-1} \Gamma_i < r_1 \Gamma_{\text{sum}} < \sum_{i=j+1}^{N} \Gamma_i$$

$$\Gamma_{\text{sum}} = \sum_{i=1}^{N} \Gamma_i$$

Then event j is executed and the time is advanced by drawing a second uniform random number:

$$\Delta t = \frac{-\ln(r_2)}{\sum_{ij} \Gamma_{a\to i}}$$

Finally, all of the events that may have changed are recalculated. In our previous work,[2] a residence time algorithm was used, neglecting updating events that may have changed due to executed transitions. Simulation is stopped when the measured physical observable reached a steady state value within a user-defined threshold.

We start the simulation by randomly placing charges on NPs with predefined density then we switch on the KMC algorithm. The mobility is measured as:

$$\mu_e = \frac{\text{harvested charges} \times L_z}{\text{t} \times \text{total number of carriers} \times F_{\text{ext}}}$$

where L_z is the length of the simulation box in the conducting direction. As stated above, we stop the simulation once the mobility reaches steady state: typically millions of time steps are needed to reach convergence. We used periodic boundary conditions in all three Cartesian directions and the number of *harvested* charges were measured by counting the net number of electrons crossing the $z = L_z$ plane.

In the current work, we extend the previous method[2] by introducing a metallic hopping channel. Concretely, we calculate the energy difference associated with the event. If the energy difference is greater than a preselected Overlap Energy (OE), then the regular thermal hopping is used. Otherwise a metallic, non-activated form is used.

For the regular hopping, we use Miller-Abrahams (MA) thermally assisted nearest-neighbor hopping or tunnelling. Other approaches include the Marcus theory of electron transfer,[3] which also takes into account nuclear relaxation effects after the hopping, and the model developed by Nelson and Chandler that closely resembles Marcus theory.[4]

The validity and differences of these approaches have been analyzed in detail.[5,6]

$$\Gamma_{i\to j} = \begin{cases} \nu\beta_{ij}\exp\left(\frac{-\Delta E_{ij}}{k_b T}\right) & \text{if } E_i > E_j \textit{ and } |\Delta E_{ij}| > OE, \\ \nu\beta_{ij} & \text{if } E_i < E_j \textit{ and } |\Delta E_{ij}| > OE, \\ \nu\beta_{ij}\exp\left(\frac{-\Delta E_{ij}}{k_b T}\right) + \nu'\Delta V & \text{if } E_i > E_j \textit{ and } |\Delta E_{ij}| <= OE, \\ \nu\beta_{ij} + \nu'\Delta V & \text{if } E_i < E_j \textit{ and } |\Delta E_{ij}| <= OE. \end{cases} \tag{1}$$

The attempt frequency ν and the metallic transition prefactor ν' are assumed to be size and ligand independent and they set the time scale of the simulations. We chose ν in order to qualitatively match the order of magnitude mobitilies measured by the Matt Law group.[7] ν', however, is chosen such that the metallic transition is at least twice as fast as the regular hopping for a typical charge transfer path.

Here, ν' is assumed to be temperature independent, but in principle it could have temperature dependence similar to the mobility of the parent bulk material. ΔV is the potential difference induced by external field between source and target NPs. β is tunnelling amplitude and we evaluate it in the WKB approximation:

$$\beta_{ij}(E) = \exp\left(-2\Delta x\sqrt{\frac{2m^*(E_{\text{vac}} - E^{tunnelling})}{\hbar^2}}\right) \tag{2}$$

Here Δx is the NP-NP surface-to-surface distance, which in practice is chosen to be twice the ligand length. m^* is the effective mass of the tunnelling medium, which also depends on the effective mass of the barrier. Here we approximated m^* with the effective masses of electrons and holes in bulk PbSe.[8] An alternative approach is to use the Bardeen formula of tunnelling.[9] Here we refer everything to the vacuum level E_{vac} which is thus set to zero in all simulations. If the NP solid was embedded in a matrix this would represent the conduction band minimum of the embedding matrix. $E^{tunnelling}$ is the tunnelling energy. It is not immediately clear what energy should be used for $E^{tunnelling}$. In the spirit of the thermally assisted hopping approach of Chandler & Nelson, E^{sp} was defined as an average of the energies of initial and final states of the hopping: $E^{tunnelling} = (E_a^{sp} + E_b^{sp})/2$, where

E^{sp} is the single particle energy.

The energy difference ΔE_{ab} in Eq.1 is the barrier for hopping, which can be written as:

$$\Delta E_{ab} = \Delta E_{ab}^{sp} + \Delta E_{ab}^{F} + \Delta E_{ab}^{C}, \tag{3}$$

where the first term on the RHS is the difference in single particle energies of the initial and final states of the hopping:

$$\Delta E_{ab}^{sp} = E_b^{sp} - E_a^{sp}. \tag{4}$$

We used the energies from $k\dot{p}$ perturbation theory as obtained by Kang&Wise.[10] We then applied a rigid shift to align the infinite diameter limit of the conduction band edge to the work function of bulk PbSe.[11]

The second term on RHS of Eq. 3 is the contribution from the external voltage V:

$$\Delta E_{ab}^{F} = q\frac{V}{L_z}(z_b - z_a), \tag{5}$$

where L_z is the length of the NP solid in the conducting direction and z_b and z_a are the z position of the center of the NPs. Care is exercised to make sure simulations are always in linear I-V regime. In particular, we set the external voltage so that $|E_{ab}^{F}| = 0.1kT(@30K)$. As mentioned in the main text, the disorder of the NP energies did not exactly average to zero in our samples, and thus generated an internal bias field. We eliminated this bias by always taking the pairwise average of the currents with a forward and a backward applied voltage.

Finally, the last term is due to the on-site Coulomb interaction:

$$\Delta E_{ab}^{C} = \Sigma_b^0 + (n_b + 1)\Sigma_b - (\Sigma_a^0 + n_a\Sigma_a) \tag{6}$$

where we introduced self energy, or the on-site charging energy: Σ^0 is the energy that

needs to be paid upon the load of the first charge onto the neutral NP, while Σ is the energy it takes to load each additional charge. Both of them can be written in the form of $\Sigma(d_{\text{particle}}) = q^2/2C(d_{\text{particle}})$, where is C is the self-capacitance of the NP. Some groups also include here the mutual capacitance of the array further decreasing the self-energy.[12] The capacitance can be taken to be proportional to the diameter d. This is the approach we followed in our previous work and X_C was chosen according to the work of Zunger.[13] In this work, instead, we use Delerue's model,[14] which provides a semi-analytic form for Σ and for Σ_0:

$$\Sigma^0 = \frac{q^2}{8\pi\epsilon_0 R}\left(\frac{1}{\epsilon_{\text{solid}}} - \frac{1}{\epsilon_{\text{NP}}}\right) + 0.47\frac{q^2}{4\pi\epsilon_0\epsilon_{\text{NP}}R}\frac{\epsilon_{\text{NP}} - \epsilon_{\text{solid}}}{\epsilon_{\text{NP}} + \epsilon_{\text{solid}}} \tag{7}$$

$$\Sigma = \frac{q^2}{4\pi\epsilon_0 R}\left(\frac{1}{\epsilon_{\text{solid}}} + 0.79\frac{1}{\epsilon_{\text{NP}}}\right) \tag{8}$$

We used the Maxwell–Garnett (MG) effective medium approximation[15] to compute the dielectric constant of the entire NP solid. According to MG, the dielectric constant of the solid can be approximated as

$$\epsilon_{\text{solid}} = \epsilon_{\text{ligand}}\frac{\epsilon_{\text{NP}}(1+\kappa f) - \epsilon_{\text{ligand}}(\kappa f - \kappa)}{\epsilon_{\text{ligand}}(\kappa + f) + \epsilon_{\text{NP}}(1-f)} \tag{9}$$

where κ is 2 for spherical NPs and f is the filling factor.

Determining the dielectric constant of NPs is a field on its own.[16,17] The high frequency dielectric constant of bulk PbSe is 22.9 at room temperature, while the low frequency dielectric constant is 210 at room temperature.[8] It is not immediately clear whether the dielectric constant entering Eqs. 7,8,9 should contain ionic relaxation effects or not. Furthermore, the dielectric constant of a single NP is in principle one by definition. One can usually define an effective dielectric constant if the NP is big enough[16,17] but it turns out that such models, e.g. Penn.,[18] may not necessarily work for any kind of system.[19] In order to avoid making an uncontrolled approximation we decided to use the high frequency bulk dielectric constant

of PbSe in the entire NP diameter range.[13]

Having calculated the total energy difference ΔE_{ab}, we then compare it against a preselected Overlap Energy (OE). If ΔE_{ab} is greater than OE, the hopping is considered to be an activated one and rate is calculated as shown in Eq.1.

Having defined the energetics of the NPs we can now discuss the transition, or hopping rates. The probability of an electron transferring from the initial NP a to the NP b is:

$$\Gamma_{ab} = \sum_{ij} \Gamma_{ij} g_i f_i(n_a) g_j (1 - f_j(n_b)) \tag{10}$$

where i/j denote kinetic energy levels, g_i/g_j are their degeneracy and f_i/f_j is the Fermi occupation function, n is the number of electrons on the respective nanoparticle.

Since we are assuming that the NP solid is weakly charged and the average charge per nanoparticle is less than the band degeneracy, the Fermi occupation functions can be replaced by their zero temperature limit and the sum over bands is limited to the first states:

$$\Gamma_{ab} = \sum_{\substack{i \in occ \\ j \in \text{unocc}}} \Gamma_{ij} g_i g_j \tag{11}$$

Following earlier works and assuming that we our NP solid is not operating in the extremely confined size regime, the degeneracy g of the band edge states comes from the valley-degeneracy of bulk PbSe which is eight, including spin. Later work of Delerue showed that there is minor split of these states due to intervalley coupling.[20]

The above described KMC framework was coded in Cython. Random numbers were generated with the Mersenne Twist Pseudorandom Number Generator.[21]

We also need to define our NP solid. The most common approach is to build a cubic lattice of NPs. This may already well capture the physics as was shown in several cases.[2,9,22] However, (i) in experiments highly monodisperse NPs tend to form denser fcc or hexagonal lattices (ii) the mobility may be underestimated due to the underestimation of the number of nearest neighbors, (iii) since most colloidal NPs have a substantial size dispersion cubic

lattices of size-disordered NPs, with NPs clamped to ideal lattice positions, may contain unrealistic voids.

In order to investigate more realistic lattice disordered NP solids we set up random closed packed models by using an event-driven Molecular Dynamics code.[23,24]

In either case, NP diameters (d) were drawn from a Gaussian distribution with an average diameter μ and standard deviation of σ:

$$f(d,\mu,\sigma) = \frac{1}{\sigma\sqrt{2\pi}} e^{-\frac{(d-\mu)^2}{2\sigma^2}} \tag{12}$$

Table S1: Parameters of the KMC framework.

Name	Value	Reference	Equation
electron effective mass, m^*	0.05	Ref.[25]	eq. (2)
NP diameter (nm)	4.0 ±0.2	NA	implicitly in eqs. (1) to (4)
ligand length (nm), $\Delta x/2$	0.5	Ref.[7]	eq. (2)
ligand dielectric constant, ϵ_{ligand}	2.0	Ref.[7]	eqs. (7) to (9)
NP dielectric constant, ϵ_{NP}	22	Ref.[8]	eqs. (7) to (9)
attempt frequency, ν(Hz)	10^{11}	see text	eq. (1)
metallic rate prefactor, ν'(Hz/V)	10^{14}	see text	eq. (1)

In order to sufficiently capture disorder effects we averaged over one hundred different random NP lattices. Error bars in our calculations represent the standard deviation of the mean. Table S1 summarizes the key parameters of the methodology used in our study. Lengths and widths of the samples were chosen to sufficiently capture disorder effects. In particular, in our earlier study we found that it is necessary to include at least 50 sites in the conducting direction.[2] We typically studied systems with a thickness of 4 layers. The lateral transverse direction was not particularly wide because generating the sample for larger sizes was a limiting factor for the code used to generate samples with high-packing density. We often used 4 NP wide samples. We chose the dimensions of our size disordered models accordingly. However, it was increasingly hard to generate reliable packing of NP solids and run the KMC simulations while keeping the density the same thus we halved the distance in the conducting direction for the charge transport calculations in the paper. However, in

order to explore the size dependence of the percolation probability, we also generated 100 samples of NP solids with 800 NPs and determined the percolation probability in both sets of samples with a Union-Find algorithm. The results are shown in Fig. S1.

Figure S1: Percolation probability, averaged over 100 samples with 400 NPs, and with 800 NPs.

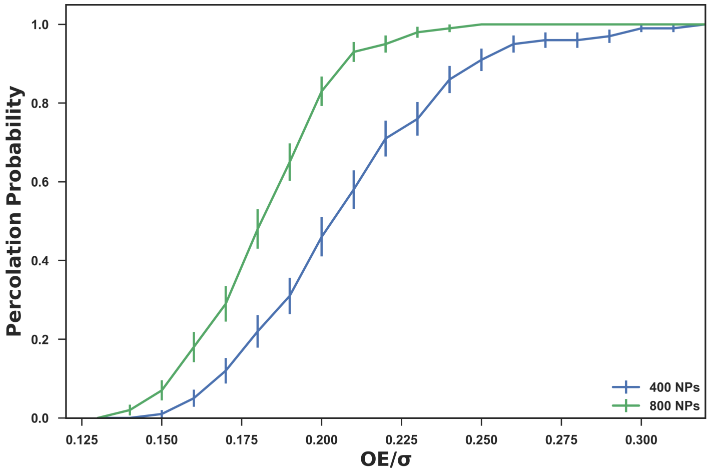

When comparing Fig. S1 to Fig. 1 in the paper with 400 NPs, we see that the threshold for the onset of percolation showed only minimal size dependence. This onset of the percolation transition was the key parameter used in critically analyzing the type of the MIT: see the dotted line in Figure 5, and therefore is one indicator that the size dependence of the conclusions of our analysis may be limited.

In the last column of the Table S1 we also showed the packing density of the size-, and lattice-disordered models. Let us recall that in the case of monodisperse spheres cubic lattice is the loosest packing with packing density of ≈ 0.52. The densest monodisperse packing (with coordination number of 12) has a packing density of ≈ 0.74. Packing densities in between are associated with loose or the mentioned close random packing. The simulations presented in the paper were obtained for samples with an average packing density of 0.64 and 100 random samples.

References

(1) Bortz, A.; Kalos, M.; Lebowitz, J. *Journal of Computational Physics* **1975**, *17*, 10 – 18.

(2) Carbone, I.; Carter, S. A.; Zimanyi, G. T. *Journal of Applied Physics* **2013**, *114*, 193709.

(3) Marcus, R. A. *The Journal of Chemical Physics* **1956**, *24*, 966–978.

(4) Chandler, R. E.; Houtepen, A. J.; Nelson, J.; Vanmaekelbergh, D. *Physical Review B* **2007**, *75*, 085325.

(5) Stephan, J.; Schrader, S.; Brehmer, L. *Synthetic Metals* **2000**, *111112*, 353–357.

(6) Vukmirovi, N.; Wang, L.-W. *Applied Physics Letters* **2010**, *97*, 043305.

(7) Liu, Y.; Tolentino, J.; Gibbs, M.; Ihly, R.; Perkins, C. L.; Liu, Y.; Crawford, N.; Hemminger, J. C.; Law, M. *Nano letters* **2013**, *13*, 1578–1587.

(8) Madelung, O., Rssler, U., Schulz, M., Eds. *Non-Tetrahedrally Bonded Elements and Binary Compounds I*; Landolt-Brnstein - Group III Condensed Matter; Springer Berlin Heidelberg, 1998; Vol. 41C; pp 1–10.

(9) Lepage, H. Modélisation de solides à nanocristaux de silicium. Ph.D. thesis, institut national des sciences appliquées de Lyon, 2013.

(10) Kang, I.; Wise, F. W. *Journal of the Optical Society of America B* **1997**, *14*, 1632–1646.

(11) Jasieniak, J.; Califano, M.; Watkins, S. E. *ACS Nano* **2011**, *5*, 5888–5902.

(12) Liu, Y.; Gibbs, M.; Puthussery, J.; Gaik, S.; Ihly, R.; Hillhouse, H. W.; Law, M. *Nano Letters* **2010**, *10*, 1960–1969.

(13) An, J. M.; Franceschetti, A.; Zunger, A. *Physical Review B* **2007**, *76*, 045401.

(14) Delerue, C.; Lannoo, M.; Allan, G. *Phys. Rev. Lett.* **2000**, *84*, 2457–2460.

(15) Merrill, W. M.; Diaz, R.; LoRe, M.; Squires, M.; Alexopoulos, N. *Antennas and Propagation, IEEE Transactions on* **1999**, *47*, 142–148.

(16) Wang, L.-W.; Zunger, A. *Phys. Rev. Lett.* **1994**, *73*, 1039–1042.

(17) Cartoixà, X.; Wang, L.-W. *Phys. Rev. Lett.* **2005**, *94*, 236804.

(18) Penn, D. R. *Phys. Rev.* **1962**, *128*, 2093–2097.

(19) Pan, D.; Wan, Q.; Galli, G. *Nature communications* **2014**, *5*.

(20) Allan, G.; Delerue, C. *Physical Review B* **2004**, *70*, 245321.

(21) Mtwister. `http://www.math.sci.hiroshima-u.ac.jp/~m-mat/MT/emt.html`, Accessed: 2016-08-25.

(22) Lepage, H.; Kaminski-Cachopo, A.; Poncet, A.; le Carval, G. *The Journal of Physical Chemistry C* **2012**, *116*, 10873–10880.

(23) Donev, A.; Torquato, S.; Stillinger, F. H. *Journal of Computational Physics* **2005**, *202*, 737 – 764.

(24) Donev, A.; Torquato, S.; Stillinger, F. H. *Journal of Computational Physics* **2005**, *202*, 765 – 793.

(25) Madelung, O., Rssler, U., Schulz, M., Eds. *Non-Tetrahedrally Bonded Elements and Binary Compounds I*; Landolt-Brnstein - Group III Condensed Matter; Springer Berlin Heidelberg, 1998; Vol. 41C; pp 1–4.
